# Non-traumatic Acute Epidural Hematoma in Multiple Sclerosis Treated With Fingolimod

**DOI:** 10.3389/fneur.2019.00763

**Published:** 2019-07-19

**Authors:** Ryoko Fukai, Keita Takahashi, Hiroyuki Abe, Yuichi Higashiyama, Hiroshi Doi, Hideyuki Takeuchi, Fumiaki Tanaka

**Affiliations:** ^1^Department of Neurology and Stroke Medicine, Graduate School of Medicine, Yokohama City University, Yokohama, Japan; ^2^Department of Neurosurgery, Graduate School of Medicine, Yokohama City University, Yokohama, Japan

**Keywords:** epidural hematoma, fingolimod, multiple sclerosis, sphingosine-1-phosphate receptor, vascular disruption

## Abstract

Fingolimod acts as a functional antagonist of the sphingosine-1-phosphate receptor and is widely used for relapsing-remitting multiple sclerosis (MS). Here we report the first case of non-traumatic acute epidural hematoma in a relapsing-remitting MS patient treated with fingolimod. Fingolimod might increase the risk of hemorrhage by enhancing vasospasm and causing vascular disruption. Switching fingolimod to other disease-modifying drugs, including dimethyl fumarate, should be considered when non-traumatic hemorrhage is observed in MS patients.

## Introduction

Fingolimod, a functional antagonist of the sphingosine-1-phosphate (S1P) receptor, is a widely used oral drug for treating relapsing-remitting multiple sclerosis (RRMS) (1). There are five S1P receptor subtypes: S1P_1_, S1P_2_, S1P_3_, S1P_4_, and S1P_5_. Fingolimod binds to S1P_1_, S1P_3_, S1P_4_, and S1P_5_, and its highest affinity is to S1P_1_ (S1P_1_ > S1P_5_ > S1P_3_ = S1P_4_) (1, 2). After binding to S1P receptors (mainly S1P_1_) on the surface of lymphocytes, fingolimod suppresses lymphocyte egress from lymphoid tissues, which prevents these cells from invading the central nervous system. Here we report the first case of non-traumatic acute epidural hematoma in a RRMS patient treated with fingolimod. Our report offers insight into the pathogenesis and risk of hematoma induced by fingolimod treatment.

## Case Report

**Figure 1:**
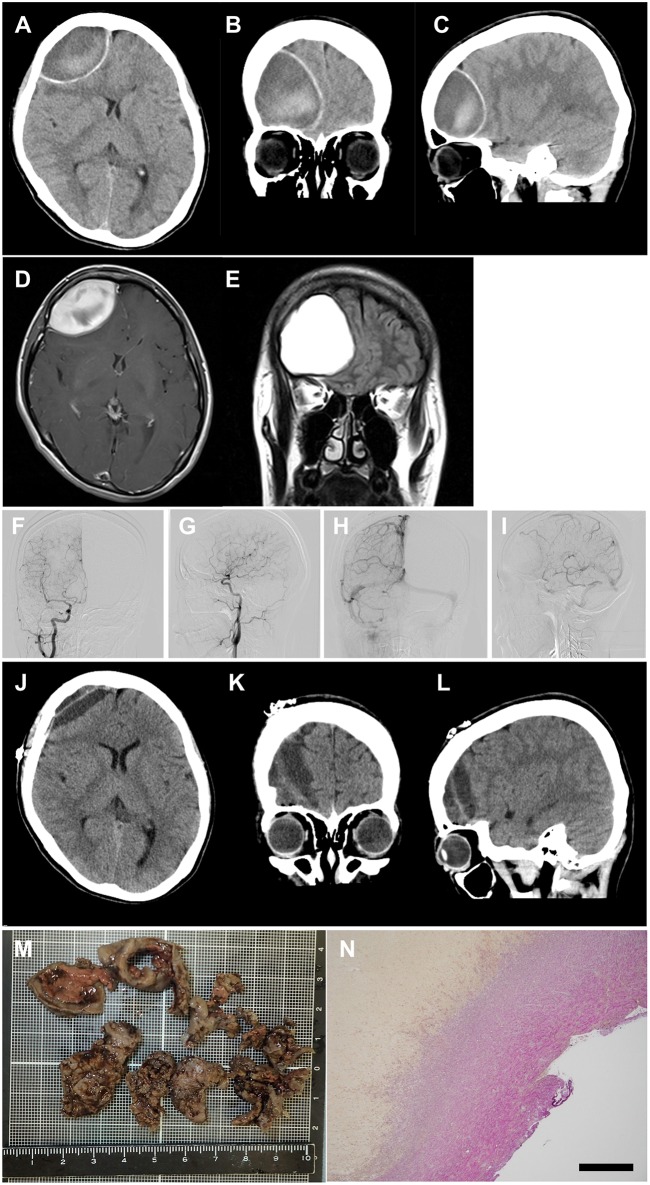
Images of acute subdural hematoma. **(A–C)** CT scan images showing an acute epidural hematoma in the right frontal lobe with retraction of the lateral ventricle anterior horn and herniation of the falx cerebri. **(D,E)** post-contrast MRI images showing neither vascular malformation nor brain tumor. **(F–I)** cerebral angiography showing neither vascular malformation nor aneurysm [**(F,G)** arterial phase; **(H,I)** venous phase]. **(J–L)** CT scan images obtained 7 days postoperatively showing successful removal of the hematoma. **(M)** macroscopic image of the removed hematoma. **(N)** microscopic image of the hematoma with Elastica van Gieson staining, demonstrating common pathological features of organized hematoma without vascular malformation or aneurysm. Scale bar, 500 μm.

A 27-year-old woman with a 3-year history of RRMS was treated with 0.5 mg/day of fingolimod for 10 months without clinical or radiological relapse. She then experienced a sudden severe headache in the forehead with no abnormal neurological signs, and was admitted to our university hospital. Brain computed tomography (CT) revealed a large epidural hematoma in the right frontal lobe with midline shift (Figures 1A–C). She had no past history of trauma or cardiovascular risk factors, including hypertension, hyperlipidemia, smoking, diabetes mellitus, collagen diseases, alcoholism, and drug abuse. In addition, radiological assessments using magnetic resonance imaging (MRI) and cerebral angiography detected no vascular malformation, aneurysm, or brain tumor (Figures 1D–I). Peripheral blood and coagulation tests exhibited no abnormalities except for mild lymphopenia (481.6 cells/μl) due to fingolimod. Fingolimod was suspected as the cause of the non-traumatic acute epidural hematoma in this case. The patient discontinued fingolimod and a month later the lymphopenia had resolved (1,131 cells/μl), but the hematoma showed no improvement. Therefore, we performed direct evacuation and successfully removed a 12 g organized hematoma (Figures 1J–M). Histological analysis disclosed common findings of organized hematoma, but no evidence of vascular disruption, vascular malformation, or malignancy (Figure 1N). After the operation, the patient received a single course of methylprednisolone pulse therapy (1 g/day for 3 successive days) followed by treatment with dimethyl fumarate instead of fingolimod. Thus, far, the patient has shown no recurrence of hematoma or bleeding for more than a year.

## Discussion

This is the first report documenting non-traumatic acute epidural hematoma in a RRMS patient treated with fingolimod. Common causes of non-traumatic epidural hematoma are bleeding secondary to coagulopathy and vascular rupture due to aneurysm or arterial–venous malformation, all of which were excluded in our case. Previous studies reported that fingolimod caused posterior reversible encephalopathy syndrome and arterial vasospasm (3, 4). S1P_1−3_ are known to be expressed on endothelial and vascular smooth muscle cells in the cardiovascular system, central nervous system, and immune systems, and regulate endothelial barrier function and peripheral vascular tone (2, 5). S1P_1_ and S1P_3_ activate endothelial nitric oxide synthase and enhance nitric oxide generation in the endothelium, leading to vasodilation secondary to smooth muscle cell relaxation (2). In addition, downregulation of S1P_1_ upregulates S1P_2_, and S1P_3_, which in turn activate Rho-kinase signaling in vascular smooth muscle cells, resulting in vasoconstriction (5–7). Vasoconstriction induces not only ischemic brain disease but also brain hemorrhage, as reported in a recent cohort study that found that 43% of patients with reversible cerebral vasoconstriction syndrome developed intracerebral hemorrhage (8). Furthermore, MS patients reportedly exhibit vascular abnormalities such as endothelial cell damage, venous drainage impairment, and cerebral hemorrhage (9, 10). Taken together, MS patients are potentially vulnerable to vascular damage, and antagonization and internalization of S1P_1_ and S1P_3_ by fingolimod may induce hemorrhage by enhancing vasospasm and/or vascular disruption. On the contrary, recent studies demonstrated that fingolimod reduced hemorrhagic transformation by protection of the blood-organ barrier integrity in rodent models of brain ischemia, intracerebral hemorrhage, and hemorrhagic shock–induced multiple organ dysfunction syndrome (11–13). In the inflamed vascular condition seen in the above models, fingolimod may have effectively suppressed endothelial cell activation. However, our patient had never shown any sign of inflammation during fingolimod treatment, which might account for the discrepant vasoreactivity to fingolimod.

In conclusion, our case report suggests that administration of fingolimod might increase the risk of brain hemorrhage by blockade of the S1P_1_ and S1P_3_ signaling pathways. Switching fingolimod to other disease-modifying drugs, including dimethyl fumarate, should be considered in MS patients with a recent history of non-traumatic brain hemorrhage.

## Ethics Statement

No investigations or interventions were performed outside routine clinical care for this patient. Written informed consent was obtained from the patient for the publication of this case report in accordance with the Declaration of Helsinki.

## Author Contributions

RF: examination, diagnosis, and therapy of the patient and drafting the manuscript. KT, YH, and HD: examination, diagnosis, and therapy of the patient. HA: surgical operation of the patient. HT and FT: study design, supervision of data analysis, interpretation and evaluation of clinical data, and drafting the manuscript.

### Conflict of Interest Statement

HT is a Review editor of Frontiers in Neurology and Frontiers in Immunology. The remaining authors declare that the research was conducted in the absence of any commercial or financial relationships that could be construed as a potential conflict of interest.
